# TGR(mREN2)27 rats develop non-alcoholic fatty liver disease-associated portal hypertension responsive to modulations of Janus-kinase 2 and Mas receptor

**DOI:** 10.1038/s41598-019-48024-4

**Published:** 2019-08-12

**Authors:** Sabine Klein, Carola-Ellen Kleine, Andrea Pieper, Michaela Granzow, Sebastian Gautsch, Mimoun Himmit, Katharina Kahrmann, Robert Schierwagen, Frank Erhard Uschner, Fernando Magdaleno, Maria Eleni Naoum, Glen Kristiansen, Thomas Walther, Michael Bader, Tilman Sauerbruch, Jonel Trebicka

**Affiliations:** 1 Department of Internal Medicine I, Goethe University Frankfurt, Frankfurt, Germany; 2 Department of Internal Medicine I, University of Bonn, Bonn, Germany; 3 House for Experimental Therapy, University of Bonn, Bonn, Germany; 4 Institute of Pathology, University of Bonn, Bonn, Germany; 5 Department of Pharmacology and Therapeutics, University College Cork, Cork, Ireland; 6 Institute of Medical Biochemistry and Molecular Biology, University Medicine Greifswald, Greifswald, Germany; 7 Berlin Institute of Health (BIH), Berlin, Germany; 8 DZHK (German Center for Cardiovascular Research), Partner Site Berlin, Berlin, Germany; 9 Institute for Biology, University of Lübeck, Lübeck, Germany; 10 Charité-University Medicine Berlin, Berlin, Germany; 11 Max Delbrück Center for Molecular Medicine, Berlin, Germany; 12 European Foundation for the Study of Chronic Liver Failure, Barcelona, Spain; 13 Institute for Bioengineering of Catalonia, Barcelona, Spain; 14 Faculty of Health Sciences, University of Southern Denmark, Odense, Denmark

**Keywords:** Mechanisms of disease, Molecular medicine

## Abstract

Prevalence of non-alcoholic fatty liver disease (NAFLD) is increasing. Resulting fibrosis and portal hypertension, as a possible secondary event, may necessitate treatment. Overexpression of mouse renin in the transgenic rat model, TGR(mREN2)27, leads to spontaneous development of NAFLD. Therefore, we used TGR(mREN2)27 rats as a model of NAFLD where we hypothesized increased susceptibility and investigated fibrosis and portal hypertension and associated pathways. 12-week old TGR(mREN2)27 rats received either cholestatic (BDL) or toxic injury (CCl_4_ inhalation). Portal and systemic hemodynamic assessments were performed using microsphere technique with and without injection of the Janus-Kinase 2 (JAK2) inhibitor AG490 or the non-peptidic Ang(1-7) agonist, AVE0991. The extent of liver fibrosis was assessed in TGR(mREN2)27 and wild-type rats using standard techniques. Protein and mRNA levels of profibrotic, renin-angiotensin system components were assessed in liver and primary hepatic stellate cells (HSC) and hepatocytes. TGR(mREN2)27 rats developed spontaneous, but mild fibrosis and portal hypertension due to the activation of the JAK2/Arhgef1/ROCK pathway. AG490 decreased migration of HSC and portal pressure in isolated liver perfusions and *in vivo*. Fibrosis or portal hypertension after cholestatic (BDL) or toxic injury (CCl_4_) was not aggravated in TGR(mREN2)27 rats, probably due to decreased mouse renin expression in hepatocytes. Interestingly, portal hypertension was even blunted in TGR(mREN2)27 rats (with or without additional injury) by AVE0991. TGR(mREN2)27 rats are a suitable model of spontaneous liver fibrosis and portal hypertension but not with increased susceptibility to liver damage. After additional injury, the animals can be used to evaluate novel therapeutic strategies targeting Mas.

## Introduction

Due to lifestyle changes, prevalence of non-alcoholic fatty liver disease (NAFLD) is increasing worldwide^1^. Only a small percent of the NAFLD patients, especially those with hepatic steatosis, will develop more severe forms of hepatic disease (fibrosis, cirrhosis). Therefore, steatosis, fibrosis and the development of portal hypertension are the principal parameters that indicate the necessity for treatment^2,3^. NAFLD is induced by various mechanisms, some of which may be modulated by drugs. However, substances acting directly on fibrogenesis and portal hypertension are rare^4^. Thus, blunting the activation of hepatic stellate cells (HSC) represents an appropriate target^5^. Fibrosis and deposition of extracellular matrix (ECM)^6^ increases portal blood outflow resistance^7^. In addition, activated HSC are hyperresponsive to vasoconstrictors (e.g. angiotensin II), which further augments hepatic resistance to portal flow^8,9^. Finally, as development of portal hypertension is associated with morbidity and mortality^10,11^, adequate therapies are important. In view of the above-mentioned demographic changes, new animal models are necessary for preclinical drug testing.

TGR(mREN2)27 is such a model, comprising the overexpression of mouse renin in transgenic rats, which leads to spontaneous development of NAFLD^12^, and underlining the pathogenic role of the renin-angiotensin system (RAS) in liver disease^11^. The role of RAS in portal hypertension is ambiguous. There exist two main axes within RAS: the classic pathway with angiotensin-converting enzyme (ACE), angiotensin (Ang) II, Ang-II-type-1 receptor (AT1R) mediating vasoconstriction - and the alternative pathway, whereby ACE2, Ang1-7, and the G-protein-coupled receptor Mas (MasR) mediate relaxation. Ang II, the agonist of the pro-fibrotic and pro-contractile AT1R, exerts its effects through Janus-Kinase 2 (JAK2) in HSC^8,9^. In the alternative RAS, ACE2 converts Ang II to Ang-(1-7), the agonist for MasR, inducing vasorelaxation mainly through nitric oxide synthase^13^. The alternative RAS has been also shown to exert beneficial effects in cirrhosis^14–16^. Interestingly, renin sets off the cascade for both, the classic and the alternative RAS, by mediating the generation of Ang I from angiotensinogen, which is further converted to Ang II by ACE, and can be further converted to Ang-(1-7) through the enzymatic activity of ACE2. Inhibition of renin, which is upstream of both pathways, leads to improved liver fibrosis and portal hypertension, suggesting a preponderance of the classic AT1R mediated pathway^17–22^. At the same time, overexpression of Ang II induces NAFLD in TGR(mREN2)27 rats^12^, a transgenic rat model with mouse renin overexpression. To date in these model fibrosis and portal hypertension has not been described. However, in humans, the renin concentration can be significantly increased by treatment with AT1R-blockers^23,24^. Since liver injury may be combined with renin overexpression^25,26^, but is absent in most animal models we used TGR(mREN2)27 rats as a model of renin-induced NAFLD. After inducing additional liver injury (BDL, CCl_4_) in TGR(mREN2)27 rats, we investigated whether these animals are more prone to fibrosis and portal hypertension.

## Results

### TGR(mREN2)27 rats develop spontaneous fibrosis and portal hypertension, but are not more prone to cholestatic or toxic liver damage

**Figure 1:**
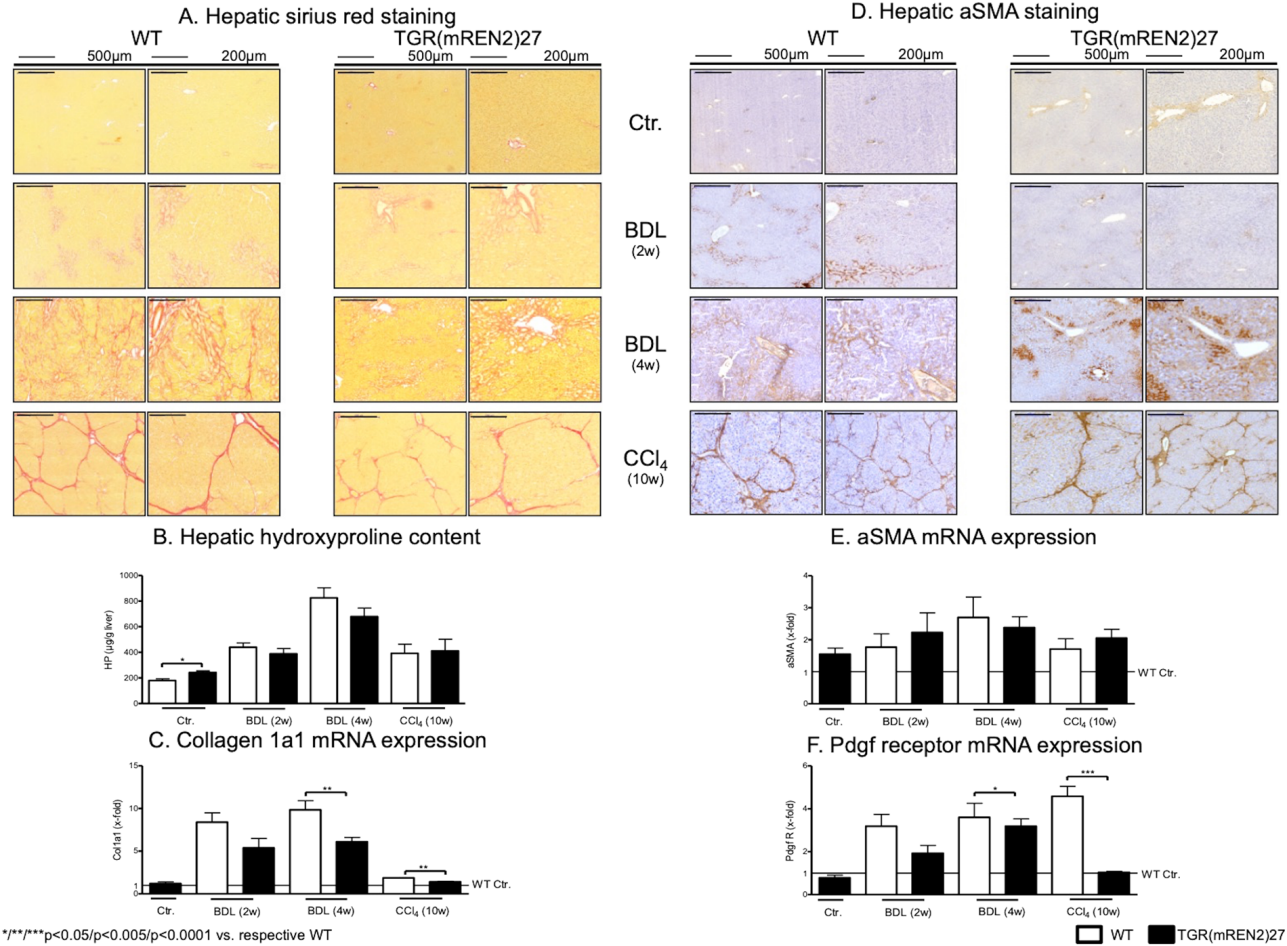
Characterization of healthy and fibrotic TGR(mREN2)27 rats. (**A**) Sirius red staining in livers of healthy and injured (BDL 2w, BDL 4w, CCl_4_ 10w) WT and TGR(mREN2)27 rats. Scales represent 500 µm and 200 µm. (**B**) Hepatic hydroxyproline content of uninjured and injured WT and TGR(mREN2)27 rats. Results are illustrated in µg/g liver. (**C**) Collagen 1a1 mRNA expression in WT and TGR(mREN2)27 livers. Expression of Col1a1 mRNA in WT controls is represented as a line. Results are expressed as x-fold change normalized to WT controls. (**D**) Hepatic aSMA immunoreactivity pictures to represent activated HSC. aSMA was performed in healthy and injured (BDL 2w, BDL 4w, CCl_4_ 10w) WT and TGR(mREN2)27 rats. Scales represent 500 µm and 200 µm. (**E**) Hepatic aSMA mRNA expression in WT and TGR(mREN2)27 rats. The mRNA expression of aSMA in WT control livers is represented as a line. Results are illustrated as x-fold normalized to WT control data. (**F**) Hepatic Pdgf receptor mRNA expression in WT and TGR(mREN2)27 rats. All results are expressed as x-fold change and were normalized to WT control livers which is illustrated as a line. (**A**–**F**) Error bars are means ± s.e.m. Statistical analyzes; Mann-Whitney t-test. ^*/**/***^Indicates p < 0.05/p < 0.005/p < 0.0001. TGR(mREN2)27 data were compared only to respective WT data.

The extent of fibrosis was assessed in TGR(mREN2)27 and wild type (WT) rats, uninjured or subjected to BDL and CCl_4_ (Fig. 1). Healthy uninjured TGR(mREN2)27 rats show more positive hepatic Sirius red staining compared to respective WT rats. There was no significant difference between the extent of fibrosis in Sirius red staining in WT and TGR(mREN2)27 rats after injury (Fig. 1A, Suppl. Fig. 1A). Interestingly, in uninjured TGR(mREN2)27 rats, periportal fibrosis was observed when compared to WT rats (Fig. 1A). Levels of hydroxyproline were significantly higher in TGR(mREN2)27 rats than in WT rats without injury (Fig. 1B). Control TGR(mREN2)27 and WT animals showed similar collagen I A I (Col1a1) mRNA levels. While there was only a trend towards decreased collagen transcription in TGR(mREN2)27 rat livers two weeks after BDL, the difference to wildtype rats became significant after four weeks of BDL. Congruent to this finding, ten weeks of CCl_4_ intoxication, TGR(mREN2)27 rats showed less hepatic Col1a1 mRNA expression than the respective WT rats (Fig. 1C). While healthy TGR(mREN2)27 rats had more positive alpha smooth muscle actin (aSMA) areas, marker of activation of hepatic stellate cells, in livers compared to respective WT livers, the hepatic aSMA increased much less after CCl_4_ intoxication in TGR(mREN2)27 rats compared to CCl_4_ intoxicated WT livers (Fig. 1D, Suppl. Fig. 1B). This was also confirmed by the total protein levels of aSMA (Suppl. Fig. 1C), but mRNA levels showed no differences between WT and TGR(mREN2)27 livers at baseline and after pathological stimuli (Fig. 1E). Already two weeks after BDL, hepatic platelet derived growth factor (PDGF)-beta receptor transcription showed a trend towards lower mRNA levels in TGR(mREN2)27 rat livers, reaching significance four weeks after BDL and in the CCl_4_ model (Fig. 1F).

TGR(mREN2)27 rats showed spontaneous portal hypertension, due to increased portal pressure and hepatic vascular resistance (Table 1). However, after four weeks of BDL injury, the degree of portal hypertension in TGR(mREN2)27 rats was similar to that found in WT rats, while CCl_4_-injury produced even less portal hypertension in TGR(mREN2)27 rats due to decreased hepatic vascular resistance compared to WT rats (Table 1). While mean arterial pressure (MAP) was higher in CCl_4_ intoxicated TGR(mREN2)27 rats compared to WT rats, systemic vascular resistances were not significantly different (Table 1).

**Table 1:** Hemodynamics in healthy and fibrotic TGR(mREN2)27 rats.

Groups		Portal pressure (mmHg)	Hepatic-vascular resistance (mmHg*min*100 g/ml)	Splanchnic-vascular resistance (mmHg*min*100 g/ml)	Systemic-vascular resistance (mmHg*min*100 g/ml)	Mean arterial pressure (mmHg)
Ctr.	WT	7.11 ± 0.37	2.14 ± 0.33	50.26 ± 7.09	11.58 ± 2.74	118.94 ± 4.11
	TGR(mREN2)27	11.24 ± 0.53^***^	5.22 ± 1.07^**^	49.92 ± 11.12	4.68 ± 0.89^*^	113.64 ± 6.68
BDL (4 w)	WT	18.47 ± 1.43	10.33 ± 3.49	31.62 ± 9.71	1.92 ± 0.72	95.47 ± 5.64^**^
	TGR(mREN2)27	14.20 ± 0.93^*^	10.50 ± 1.39	22.32 ± 6.20	2.34 ± 0.38^***^	89.03 ± 5.41^***^
CCl_4_ (10w)	WT	22.56 ± 2.43	13.27 ± 2.43	20.55 ± 4.40	2.08 ± 0.68	91.00 ± 4.39
	TGR(mREN2)27	17.8 ± 0.92^***^	9.80 ± 2.43^*^	43.40 ± 8.35^*^	2.49 ± 0.21	109.60 ± 5.47^*^

^*/**/***^p < 0.05/p < 0.005/p < 0.0001 vs. respective WT.

### Expression of RAS components in TGR(mREN2)27 rats

**Figure 2:**
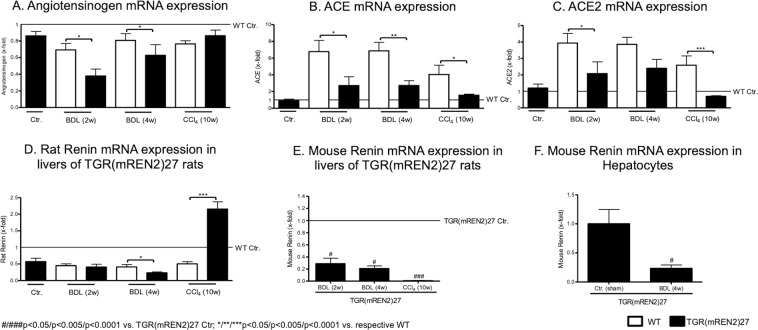
Hepatic gene expression levels of the renin-angiotensin system in healthy and cirrhotic TGR(mREN2)27 rats. (**A**) Hepatic angiotensinogen mRNA expression in control, BDL (2w, 4w) and CCl4 (10w) intoxicated WT and TGR(mREN2)27 rats. All results were normalized to WT control data, which is shown as a line. Results are expressed as x-fold change. ACE mRNA expression in livers of WT and TGR(mREN2)27 rats. Results of uninjured and injured (BDL 2w, BDL 4w, CCl_4_ 10w) rats are expressed as x-fold change to WT control data, which is illustrated as a line. mRNA levels of hepatic ACE2 expressed as x-fold level of transcription normalized to WT controls, shown as a line. (**D**) Rat renin mRNA expression in livers of WT and TGR(mREN2)27 rats. Results are expressed as x-fold change and normalized to WT controls, represented as a line. (**E**) Mouse renin mRNA expression in livers of uninjured TGR(mREN2)27 rats and after BDL (2w, 4w) and CCl_4_ 10w. Results were normalized to uninjured control TGR(mREN2)27 data and expressed as x-fold change. (**F**) Mouse renin mRNA expression in hepatocytes of control (sham) and BDL (4w) TGR(mREN2)27 rats. Data are expressed as x-fold change of control TGR(mREN2)27 rats. Error bars are means ± s.e.m. Statistical analyzes; Mann-Whitney t-test. ^*/**/***^Indicates p < 0.05/p < 0.005/p < 0.0001 compared to respective WT data. ^#/##/###^Indicates p < 0.05/p < 0.005/p < 0.0001 compared to TGR(mREN2)27 control data.

Interestingly, hepatic angiotensinogen mRNA levels decreased after BDL, which was more pronounced in TGR(mREN2)27 rats (Fig. 2A). ACE and ACE2 mRNA increased after liver injury, but to a lesser extent in TGR(mREN2)27 rat livers than in WT livers (Fig. 2B,C). Moreover, hepatic rat renin mRNA expression was down-regulated after liver injury, with down-regulation most pronounced in TGR(mREN2)27 rats after four weeks of BDL. This direction of regulation was apposed in CCl_4_ TGR(mREN2)27 rat livers compared to the respective WT livers (Fig. 2D). In contrast, expression of hepatic transgene (mouse renin) was down-regulated in TGR(mREN2)27 rats (Fig. 2E), which was most likely due to the down-regulation in the hepatocytes, as shown in primary isolated hepatocytes from BDL TGR(mREN2)27 rats compared to control TGR(mREN2)27 rats (Fig. 2F).

### AT1R-dependent pathways in TGR(mREN2)27 rats

**Figure 3:**
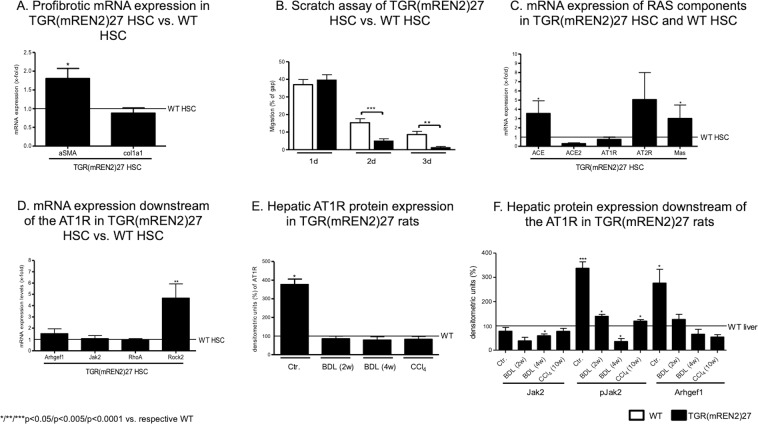
Characteristics of hepatic stellate cells in TGR(mREN2)27 rats. (**A**) aSMA and Col1a1 mRNA expression levels in TGR(mREN2)27 HSC compared to WT HSC. mRNA expression levels of WT HSC are represented as a line and were used for normalization of mRNA expression levels of TGR(mREN2)27 HSC. Results are illustrated as x-fold change. (**B**) Scratch assay of WT and TGR(mREN2)27 HSC to represent the migration after one, two and three days. Results are shown as percentage of the gap. A decreased gap represents an increased migration. (**C**) mRNA expression levels of ACE, ACE2, AT1R and Mas in HSC of TGR(mREN2)27 rats normalized to expression levels of WT HSC, illustrated by a line. Results are shown as x-fold change. (**D**) Gene expression of Arhgef1, Jak2, RhoA and Rock2 in HSC of TGR(mREN2)27 rats compared to expression levels in WT HSC, which is represented as a line. Results of mRNA expression levels in TGR(mREN2)27 HSC are normalized to WT HSC and represented as x-fold change. (**E**) Hepatic protein expression of AT1R in uninjured (control) and injured (BDL 2w, BDL 4w, CCl_4_ 10w) TGR(mREN2)27 rats normalized and compared to WT livers. mRNA expressions of WT livers are represented as a line and results are illustrated as densitometric units. (**F**) Hepatic protein expression of Jak2, pJak2 and Arhgef1 in TGR(mREN2)27 rats (control, BDL 2w, BDL 4w, CCl_4_ 10w). Bars are represented as percentage of densitometric units and compared to WT liver expression levels, shown as a line.

Since TGR(mREN2)27 rats showed no major differences compared to WT rats in the *in vivo* disease models, we next analyzed HSC as the major cellular contributor of liver fibrosis. Compared to WT HSC, HSC isolated from TGR(mREN2)27 showed higher aSMA mRNA expression without changes in collagen mRNA (Fig. 3A).

To evaluate the motility of the cells, we investigated migration using the scratch assay, in which HSC isolated from TGR(mREN2)27 showed higher migration capacity than WT HSC (Fig. 3B).

Interestingly, the mRNA levels of ACE, AT2R and Mas suggest an elevated cellular alternative RAS pathway in activated HSC of TGR(mREN2)27 rats gained from livers of uninjured TGR(mREN2)27 rats (Fig. 3C).

Although most downstream pathways activated by AT1R were similarly regulated in WT and TGR(mREN2)27 HSC, congruent to the unaltered mRNA expression of the receptor, HSC from TGR(mREN2)27 HSC showed significantly elevated ROCK2 expression (Fig. 3D). Interestingly, the total hepatic AT1R protein expression was elevated, but only on basal condition TGR(mREN2)27 rats and this difference disappeared after additional liver injury (Fig. 3E, Suppl. Fig. 1D). In parallel to this finding, JAK2 phosphorylation and its downstream component Arhgef1 were overexpressed in the control TGR(mREN2)27 rat livers when compared to WT (Fig. 3F, Suppl. Fig. 1C), a difference that could not be observed under liver injury conditions.

**Figure 4:**
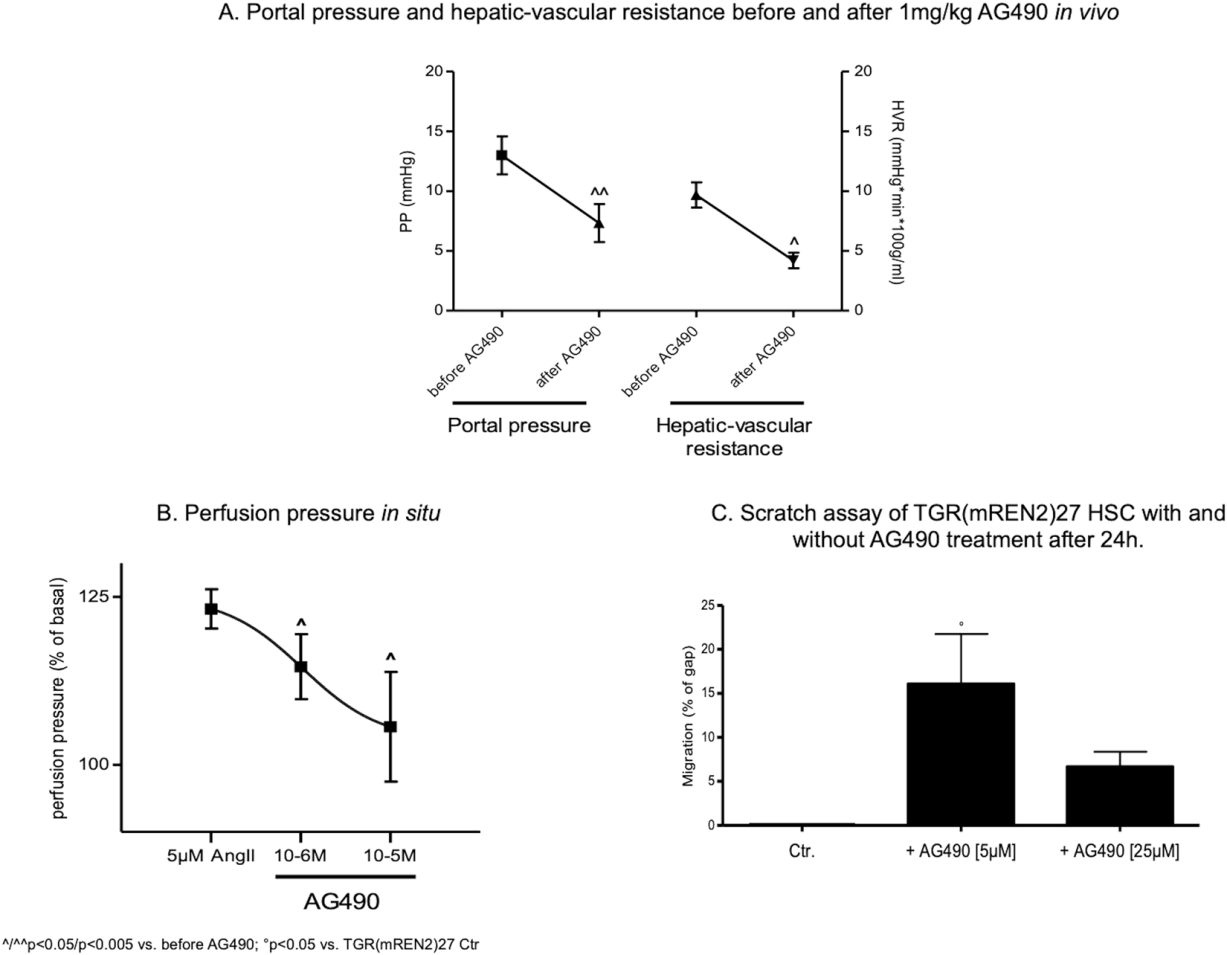
Jak2 upregulation in TGR(mREN2)27 rats. (**A**) Portal pressure and hepatic-vascular resistance before and after injection of 1 mg/kg AG490. In TGR(mREN2)27 rats, portal pressure was significantly reduced after inhibition of Jak2 by AG490. Unit of portal pressure measurement is mmHg and of hepatic-vascular resistance mmHg*min*100 g/ml. (**B**) Perfusion pressure *in situ* in livers of TGR(mREN2)27 rats after precontraction with 5 µM Ang II and after 10^−6^ and 10^−5^M AG490 in the perfusion buffer. Results are represented as percentage of the basal perfusion pressure. (**C**) Scratch assay of TGR(mREN2)27 HSC with and without AG490 treatment (5 µM and 25 µM) after 24 h. Migration of TGR(mREN2)27 HSC is represented as percentage of gap. Gap of untreated HSC are normalized to 100%. Error bars are means ± s.e.m. Statistical analyzes; Paired Wilcoxon signed rank test. ^^/^^^Indicates p < 0.05/p < 0.005 compared to measurement before AG490 treatment. ^#/###^Indicates p < 0.05/p < 0.0001 compared to TGR(mREN2)27 control data.

In order to test whether this up-regulation is relevant for the spontaneous portal hypertension observed in TGR(mREN2)27 rats (Table 1), AG490 compound was used to inhibit JAK2. Indeed, AG490 decreased portal pressure and hepatic vascular resistance in TGR(mREN2)27 rats (Fig. 4A). This effect was also confirmed by *in situ* liver perfusion experiments in TGR(mREN2)27 livers, showing a dose-dependent relaxation of the intrahepatic vascular system (Fig. 4B). Furthermore, migration of TGR(mREN2)27 HSC was blunted by AG490 (Fig. 4C).

### Role of Mas in TGR(mREN2)27 rats

**Figure 5:**
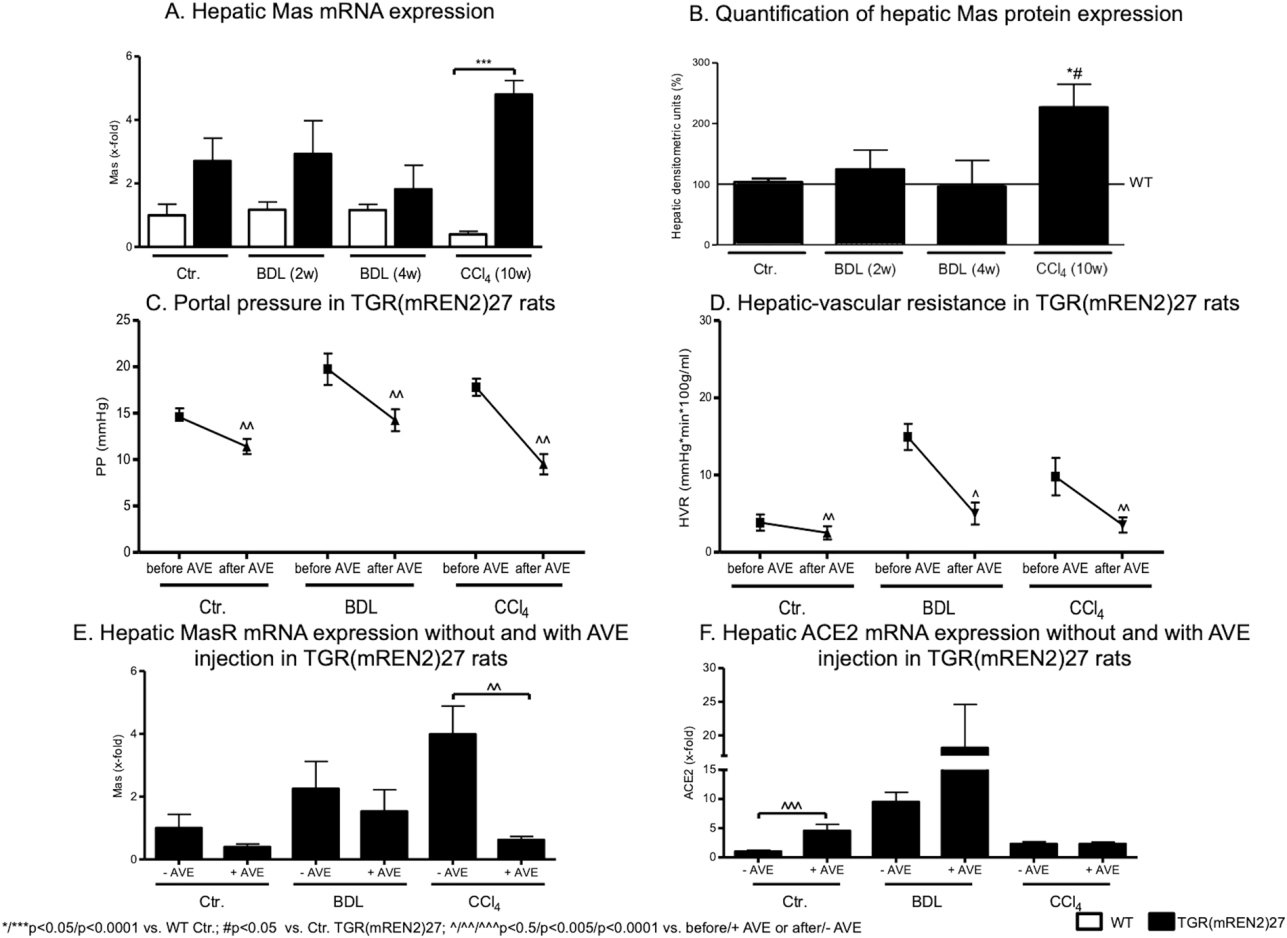
Overexpression of Mas in TGR(mREN2)27 rats. (**A**) Hepatic Mas mRNA expression in WT and TGR(mREN2)27 rats without liver injury (control) and after injury (BDL 2w, BDL 4w, CCl_4_ 10w). mRNA expression data were normalized to WT control data and illustrated as x-fold change. (**B**) Quantification of hepatic Mas protein expression. Hepatic protein expression of Mas in TGR(mREN2)27 livers (control, BDL 2w, BDL 4w, CCl_4_ 10w) compared and normalized to WT Mas protein expression data. Results are shown as percentage of densitometric units and WT data were set at 100% and illustrated as a line. (**C**) Portal pressure in TGR(mREN2)27 rats. Portal pressure was measured in mmHg before and after AVE injection in control, BDL and CCl_4_ intoxicated TGR(mREN2)27 rats. (**D**) Hepatic-vascular resistance in TGR(mREN2)27 rats. Hepatic-vascular resistance was measured in control, BDL and CCl_4_ intoxicated TGR(mREN2)27 rats before and after AVE injection. Results are shown as mmHg*min*100 g/ml. (**E**) Hepatic Mas mRNA expression with and without AVE injection in TGR(mREN2)27 rats. Hepatic Mas mRNA was measured in control, BDL and CCl_4_ injected TGR(mREN2)27 rats without and with AVE injection. Data were normalized to control mRNA data without AVE treatment and represented as x-fold change. (**F**) Hepatic ACE2 mRNA expression with and without AVE injection in TGR(mREN2)27 rats. ACE2 mRNA expression was measured in livers of control, BDL and CCl_4_ intoxicated TGR(mREN2)27 rats with and without AVE injection. All data were normalized to control data without AVE injection und shown as x-fold change. Error bars are means ± s.e.m. Statistical analyzes; Mann-Whitney t-test. ^*/**/***^Indicates p < 0.05/p < 0.005/p < 0.0001 compared to respective WT data. ^#/###^Indicates p < 0.05/p < 0.005/p < 0.0001 compared to TGR(mREN2)27 control data. Paired t-test ^^/^^/^^^^Indicates p < 0.05/p < 0.005/p < 0.0001 compared to measurement before AVE treatment.

Interestingly, hepatic Mas expression tended to be higher in TGR(mREN2)27 rats than in WT rats at mRNA and protein levels, reaching significance in CCl_4_ induced fibrosis (Fig. 5A,B, Suppl. Fig. 1D). To test whether this up-regulation of Mas is relevant *in vivo*, its agonist AVE0991 was injected in control TGR(mREN2)27 animals, as well as after four weeks of BDL and ten weeks of CCl_4_. A significant decrease of portal pressure due to a drop in hepatic vascular resistance was observed in TGR(mREN2)27 rats in all models (Fig. 5C,D, Table 2). Secondary changes in the systemic circulation were also observed. However, in splanchnic vascular resistance, they were not significant (Table 2).

**Table 2:** Hemodynamics in healthy and fibrotic TGR(mREN2)27 rats before and after AVE0991 injection.

Group		Portal pressure (mmHg)	Hepatic-vascular resistance (mmHg*min*100 g/ml)	Splanchnic-vascular resistance (mmHg*min*100 g/ml)	Mean arterial pressure (mmHg)	Systemic-vascular resistance (mmHg*min*100 g/ml)
TGR(mREN2)27 Ctr.	before AVE0991	14.6 ± 0.93	3.84 ± 1.05	29.45 ± 4.62	128.6 ± 4.04	29.45 ± 4.62
	after AVE0991	11.4 ± 0.81^**^	2.51 ± 0.85	24.43 ± 5.43	119.6 ± 6.12	24.43 ± 5.43
TGR(mREN2)27 BDL (4w)	before AVE0991	19.75 ± 1.70	14.94 ± 1.71	15.24 ± 1.65	75.5 ± 5.17	15.25 ± 1.65
	after AVE0991	14.25 ± 1.18^**^	5.01 ± 1.42^*^	27.63 ± 13.35	74.75 ± 7.00	27.63 ± 13.35
TGR(mREN2)27 CCl_4_ (10w)	before AVE0991	17.8 ± 0.92	9.80 ± 2.43	43.40 ± 8.35	109.6 ± 5.47	43.40 ± 8.35
	after AVE0991	9.5 ± 1.10^***^	3.53 ± 0.98^*^	45.37 ± 8.69	126.8 ± 8.99	45.37 ± 8.69

^*/**/***^p < 0.05/p < 0.005/p < 0.0001 vs. before AVE0991.

Interestingly, AVE0991 decreased Mas expression but led to a relative increase in ACE2 mRNA levels (Fig. 5E,F), which might be a feedback regulation to the reduced Mas mRNA expression in response to AVE0991. Notably, ACE and AT1R were not substantially influenced (Suppl. Fig. 1E,F).

## Discussion

This study shows for the first time that TGR(mREN2)27 rats develop spontaneous fibrosis and portal hypertension, in addition to previously described steatosis and inflammation^12^. Renin-induced portal hypertension can be ameliorated either by JAK2 inhibitors or Mas agonists. Surprisingly, renin overexpression does not exacerbate cholestatic or toxic liver damage.

Modulation of RAS might play an important role in the treatment of human NAFLD^27,28^, suggesting a pathogenic involvement of RAS in the development and progression of NAFLD. NAFLD is an emerging etiology of liver disease and almost 30% of the patients suffer from portal hypertension, which is associated with fibrosis in almost 90% of the patients^2^. The TGR(mREN2)27 rat model has been described to exhibit pronounced steatosis and inflammation already at an age of 12 weeks^12^ and the present study demonstrates that these animals also develop fibrosis and portal hypertension. This further confirms TGR(mREN2)27 as a suitable model to investigate renin-induced liver injury and portal hypertension. Moreover, TGR(mREN2)27 rats represent a NAFLD model of rats with mild fibrosis and significant portal hypertension regardless of any specific diet. To date, all NAFLD rat models necessitate an extended diet treatment and, while the animals might develop portal hypertension, they nevertheless fail to develop inflammation or fibrosis^29–31^. Thus, the TGR(mREN2)27 rat model possibly offers an advantage over the diet models in wild type rats^29–31^.

The concept that co-factors of liver injury may maintain portal hypertension has already been adopted by the Baveno VI guidelines^32^, and was tested in TGR(mREN2)27 rats. However, this concept could not be implemented in the rats in our study, since the mouse renin expression - responsible for the development of liver fibrosis and portal hypertension - was reduced upon cholestatic or toxic liver injury. This reduction was probably caused by the injury inducing loss of hepatocytes, which are the majority cell type in the liver. During liver injury the number of hepatocytes diminishes, and as shown by our study the gene expression of mREN is decreased in the hepatocytes from fibrotic animals. This reduced mouse renin expression due to injury might explain the lacking difference between WT and TGR(mREN2)27 rats after liver injury.

However, the TGR(mREN2)27 rats without an additional liver injury are an interesting model of liver disease since these animals develop NAFLD with fibrosis and portal hypertension. Indeed, classic and alternative RAS play an important role in different models of experimental liver cirrhosis and in different etiologies of human liver cirrhosis^8,9,14–16^. TGR(mREN2)27 rats exhibit these properties, which confirms their suitability as a model of liver disease. One of the pathways mediating portal hypertension in TGR(mREN2)27 rats is the overactivation of the JAK2-dependent pathway downstream of AT1R. Several lines of evidence confirmed this conclusion. First, the transcription levels of the main components of the pathway including AT1R were increased. Further, activation and protein expression of JAK2 and Arhgef-1, and, finally, inhibition of JAK2 using AG490 decreased hepatic resistance and thereby portal pressure in these animals. Previously, we demonstrated that downstream of AT1R, JAK2/Arhgef1 is activated and mediated through ROCK fibrosis and portal hypertension^8,9^. These findings were independently confirmed by others^33^. In uninjured TGR(mREN2)27 rats, JAK2 inhibition mediated relaxation of hepatic stellate cells, slowed their migration and thereby decreased portal pressure *in vivo* via a decrease in hepatic vascular resistance, which was shown *in vivo* and in isolated liver perfusions.

Similar to human liver cirrhosis with portal hypertension, TGR(mREN2)27 rats showed elevated Mas expression^15^. AVE0991, the non-peptidic agonist of Mas, could blunt portal hypertension due to massive hepatic vasodilation in TGR(mREN2)27 rats, in either uninjured or cirrhotic (BDL, CCl_4_) models. Again, this indicates that Mas is an important effector of the vasculature in the presence of portal hypertension as described previously by others and our group^14–16^. The assessment of the loss-of-function of the masR would be extremely interesting in this setting and this is a limitation of the present work and can be further analyzed in future studies. Especially the hemodynamic results suggest that not only uninjured TGR(mREN2)27 rats, but also TGR(mREN2)27 rats after cholestatic or toxic injury are suitable for analyzing the effects of Mas. Interestingly, especially the incubation with AVE0991 led to a decrease of masR mRNA, significantly only in the CCl4-model. This might be due to an yet unknown feedback-loop, which might also explain why AVE0991 did not reduce fibrosis in the long-term treatment.

In summary, TGR(mREN2)27 rats develop spontaneous liver fibrosis and portal hypertension and are a suitable NAFLD model with mild liver fibrosis and portal hypertension. In particular after toxic or cholestatic liver injury, may TGR(mREN2)27 rats be used to develop novel therapeutic strategies targeting Mas.

## Materials and Methods

### Animals and models of liver disease

#### Animals

We used 79 Sprague-Dawley wild type (WT) and 90 TGR(mREN2)27 rats. Experimental procedures were approved by the Animal Ethics Committee of Austin Health and of North Rhine-Westphalia (LANUV 84-02.04.2014.A137). WT and TGR(mREN2)27 rats were housed in a controlled environment (12 hour light/dark, temperature 22 °C to 24 °C) and fed standard rat chow *ad libitum* (Norco, Lismore NSW, Australia; Ssniff, Soest, Germany) with free access to water.

#### Toxic model

Eight WT and 20 TGR(mREN2)27 rats (100 g) underwent twice weekly inhalation of 1 l/min CCl_4_ for ten weeks until ascites was present as described previously^9,34^. Age-matched control rats (ten WT and ten TGR(mREN2)27) did not receive CCl_4_.

#### Cholestatic model

After one week of acclimatization, BDL was performed as previously described in 40 WT and 45 TGR(mREN2)27 rats (180 g) for four weeks^9,34^. BDL rats were compared to 21 WT and 15 TGR(mREN2)27 sham-operated rats.

### Hemodynamic studies

#### *In-vivo* hemodynamic studies

Once rats had developed ascites as a definite sign for the presence of portal hypertension, the animals were used for hemodynamic studies as described previously^9,34^. To assess the acute effect of AVE0991 or AG490, invasive measurements of mean arterial pressure (MAP) and portal pressure (PP) were performed in cirrhotic rats. AVE0991 or AG490 was administered at a dose of 1 mg/kg in the femoral vein.

#### Microsphere technique

To investigate hemodynamics, the colored microsphere technique was performed as described previously^9,34^. Before and 1 h after injection of AVE0991 or AG490, 300.000 systemic (red/white) microspheres (15 µm diameter, Triton-Technologies, San Diego, USA) were injected in the left ventricle. Mesenteric portosystemic shunt volume was estimated before and after injection of 150.000 microspheres (yellow/blue) in the ileocecal vein.

#### *In situ* liver perfusion

In ten cirrhotic CCl_4_ intoxicated TGR(mREN2)27 rats, *in situ* liver perfusion was performed in a recirculating system as described previously^9,34^. After a stabilization period of 30 minutes, pre-contraction of the liver was induced by adding 5 µM Ang II to the Krebs-Henseleit solution. Thereafter, AG490 was added to the Krebs-Henseleit solution (10^−6^ and 10^−5^ M).

#### Hepatic hydroxyproline content

The hepatic hydroxyproline content was determined photometrically in analogue segments (200 mg) of snap-frozen livers as described previously^8,35^.

### Tissue collection

Healthy control rats and animals after induction of liver fibrosis were anesthetized and laparotomy was performed for tissue collection. The livers were cut into fragments and stored at −80 °C until they were used for qRT-PCR and Western blot analysis as described previously^8,35^. Segments of each liver were fixed in formaldehyde (4%) for paraffin embedding as described previously^8,35^.

#### Histological staining

Paraffin-embedded liver sections (2–3 µm) were treated with 0.1% Sirius red in saturated picric acid (Chroma, Münster, Germany) to detect collagen fibers. Hepatic Sirius red stainings were digitalized using Pannoramic MIDI (3DHistech, Budapest, Hungary) and quantified using Histoquant (3DHistech, Budapest, Hungary) as previously described^8,35^.

### Isolation of primary hepatocytes and hepatic stellate cells

Primary rat hepatic stellate cells were isolated and cultured as previously described^8,35^. Viability and purity were routinely >95%. For early activation of HSCs, cells were harvested on day 10.

### Cell culture

Rat hepatic stellate cells were incubated in cell culture medium (DMEM + 20% FBS + penicillin/streptomycin) in 250 ml plastic flasks at 37 °C. After reaching 80% confluence, cells were passaged with a 1:3 split ratio. Detachment was achieved by incubating the cells with 0.05% Trypsin/EDTA solution (solved in PBS) for five minutes at 37 °C. Confluent hepatic stellate cells were incubated in media with 5 µM AG490 or with 10^−5^ M AVE0991 and harvested three days later for qRT-PCR.

#### Wound healing assay

Cells were cultured in cell culture medium (DMEM + 20% FBS + penicillin/streptomycin) in 24-well plastic dishes at 37 °C. When cells were confluent the cell culture medium was withdrawn by suction and changed with the respective media. A scratch was made with a size of 1 mm. After one, two, and three days, the diameter of the scratch was measured and quantified using a Zeiss microscope (Primo Star, SF18). The results are shown as the percentage of the gap size.

### qRT-PCR

Hepatic stellate cells and liver homogenates from either fibrotic or non-fibrotic rats were prepared using previously described methods^8,35^. RNA isolation, reverse transcription, and detection by real-time polymerase chain reaction (RT-PCR) were performed as described previously^8,35^. RNA was isolated from samples using the Qiazol reagent as instructed by the manufacturer (Qiagen, Hilden, Germany). The following assays provided by Applied Biosystems (Foster City, USA) were used: *ACTA2* (αSMA, Rn01759928_g1), *COL1A1* (Rn00801649_g1), *Tgfb1* (*Rn00572010_m1*), *PDGFRB* (Rn00709573_m1), *Angiotensinogen* (Rn00593114_m1), ACE (Rn00561094_m1), *ACE2* (Rn01416293_m1), *AT1R* (Rn01435427_m1), *Mas* (Rn00562673_s1), *Ccl2* (Rn00580555_m1), *Emr1* (Rn01527631_m1), *Renin rat* (Rn00561847_m1), *Renin mouse* (Mm00651435_mH), *Arhgef1* (Rn00572505_m1), *Jak2* (Rn00676341_m1), *RhoA* (Rn04219609_m1) and *Rock2* (Rn00564633_m1) (was provided by Qiagen (Hilden, Germany)). Samples were normalized to 18 s rRNA.

### Western blotting

Hepatic stellate cells and liver samples were processed as previously described using sodium dodecyl sulfate polyacrylamide gel electrophoresis (SDS-PAGE) and nitrocellulose membranes. Equal protein loading was assured using Ponceau-S staining. GAPDH served as endogenous control of protein expression. Membranes were incubated with rabbit-anti- Arhgef (Arhgef1), rabbit-anti-Jak2 (Jak2) from Cell Signaling (Danvers, MA, USA), rabbit-anti-pJak2 (pJak2, Y1007/1008), rabbit-anti-AT1R (AT1), rabbit-anti-ROCK (Rock2), rabbit-anti-pMoesin (p-Moesin), rabbit-anti-Mas1 (Mas) from Santa Cruz Biotechnology (Heidelberg, Germany) and rabbit-anti-GAPDH primary antibodies and corresponding peroxidase-coupled secondary antibodies from Santa Cruz Biotechnology (Heidelberg, Germany). Results were analyzed using Chemi-Smart digital detection (PeqLab, Biotechnologies, Erlangen, Germany) after enhanced chemiluminescence (ECL, Amersham, UK).

### Statistical analysis

Group size was at least n = 5 for each group. Graphs are presented as means ± standard error of the mean (SEM) and p-values < 0.05 were considered statistically significant. Western blots were measured using digital densitometry software (Bio-1D v.15.02, Vilber Lourmat, Marne-la-Vallée, France) and the respective density of each band was calculated. The fibrosis groups were tested for significance to their corresponding controls using Mann-Whitney *U* test. In qPCR experiments, 2^−ddCT^ was calculated and normalized to the respective control group. Plotting of diagrams and statistical analysis were performed using GraphPad Prism version 4.00 for Windows (GraphPad Software, La Jolla, California, USA).

All methods were performed in accordance with the relevant guidelines and regulations.

## Supplementary information

Supplementäre Figure 1
