# Supplementary material for: TGR(mREN2)27 rats develop non-alcoholic fatty liver disease-associated portal hypertension responsive to modulations of Janus-kinase 2 and Mas receptor
Source: Sci Rep. 2019 Aug 12;9:11598. doi: 10.1038/s41598-019-48024-4 (PMC6690919; doi:10.1038/s41598-019-48024-4)
Supplement: Supplementary file 1 — Supplementäre Figure 1 [file 41598_2019_48024_MOESM1_ESM.pdf]

# TGR(mREN2)27 rats develop non-alcoholic fatty liver disease-associated portal hypertension responsive to modulations of Janus-kinase 2 and Mas receptor

Sabine Klein[1], Carola-Ellen Kleine[2], Andrea Pieper[3], Michaela Granzow[2], Sebastian Gautsch[2], Mimoun Himmit[2], Katharina Kahrmann[2], Robert Schierwagen[1], Frank Erhard Uschner[1], Fernando Magdaleno[2], Maria Eleni Naoum[13], Glen Kristiansen[4], Thomas Walther[5, 6], Michael Bader[7, 8, 9, 10, 11], Tilman Sauerbruch[2], Jonel Trebicka[1, 12, 13, 14]

[1] Department of Internal Medicine I, Goethe University Frankfurt, Frankfurt, Germany

[2] Department of Internal Medicine I, University of Bonn, Bonn, Germany

[3] House for Experimental Therapy, University of Bonn, Bonn, Germany

[4] Institute of Pathology, University of Bonn, Bonn, Germany

[5] Department of Pharmacology and Therapeutics, University College Cork, Cork, Ireland

[6] Institute of Medical Biochemistry and Molecular Biology, University Medicine Greifswald, Greifswald, Germany

[7] Berlin Institute of Health (BIH), Berlin, Germany

[8] DZHK (German Center for Cardiovascular Research), Partner Site Berlin, Berlin, Germany

[9] Institute for Biology, University of Lübeck, Lübeck, Germany

[10] Charité-University Medicine Berlin, Germany

[11] Max Delbrück Center for Molecular Medicine, Berlin, Germany

[12] European Foundation for the Study of Chronic Liver Failure, Barcelona, Spain

[13] Institute for Bioengineering of Catalonia, Barcelona, Spain

[14] Faculty of Health Sciences, University of Southern Denmark, Odense, Denmark

**Supplemental Figure 1:**

A) Quantification of hepatic Sirius red staining. Quantified hepatic Sirius red stainings represented in Figure 1 were analyzed using 3DHISTECH Ltd. Software. Quantified Sirius red stainings of TGR(mREN2)27 rats were compared to respective WT quantifications in control,BDL (2w, 4w) and $CCl_4$ (10w) groups. Results are expressed as percentage of positive stained area. B) Quantification of hepatic aSMA staining. Positive aSMA stainings of healthy, injured BDL (2w, 4w) and $CCl_4$ (10w) intoxicated TGR(mREN2)27 livers were compared with positive aSMA stainings in respective WT livers. Results are shown as percentage of positive stained area. C) Hepatic protein expressions downstream AT1R. Protein expression levels of Jak2, pJak2, Arhgef1 and aSMA were detected by Western blots. Each representative Western blot shows on the left hand side, the expression of hepatic WT proteins and on the right hand side, the expression of hepatic TGR(mREN2)27 proteins. Each hepatic protein expression is illustrated for sham operated, BDL (2w, 4w) and $CCl_4$ (10w) intoxicated rats. D) Hepatic protein expression of AT1R and Mas. The hepatic protein expression of AT1R and Mas were investigated by Western blot. Each Western blot shows hepatic WT protein expressions (left) and hepatic TGR(mREN2)27 protein expressions (right). Protein expressions of AT1R and Mas are shown for healthy sham operated, BDL (2w, 4w) and CCl4 (10w) intoxicated livers. E) mRNA expressions of hepatic ACE mRNA without and with AVE injection in TGR(mREN2)27 rats. Livers of TGR(mREN2)27 rats were analyzed for ACE mRNA expressions with and without AVE injection. Protein expression levels of healthy control livers and injured livers (BDL, $CCl_4$) were normalized to healthy control livers without AVE injection. Hepatic mRNA expressions are represented in x-fold change. F) Hepatic AT1R mRNA expression with and without AVE injection in TGR(mREN2)27 rats. Hepatic AT1R mRNA expression of TGR(mREN2)27 rats were normalized to control TGR(mREN2)27 values and represented as x-fold change. Bars are illustrated as means ± s.e.m. Statistical analyzes; Mann-Whitney t-test. */** indicates $p<0.05/p<0.005$ compared to respective WT or TGR(mREN2)27 controls.

# Supplemental Figure 1:

A. Quantification of hepatic sirius red staining

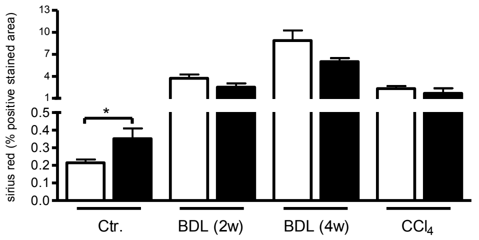


B. Quantification of hepatic αSMA staining

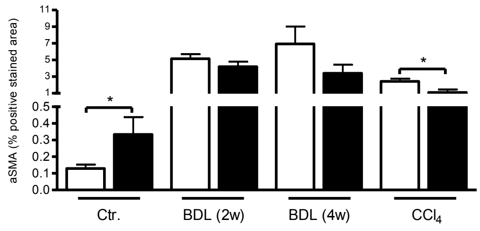


C. Hepatic protein expressions downstream AT1R

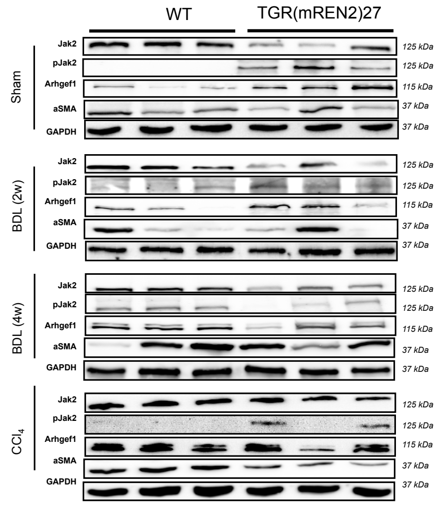


D. Hepatic protein expression of AT1R and Mas

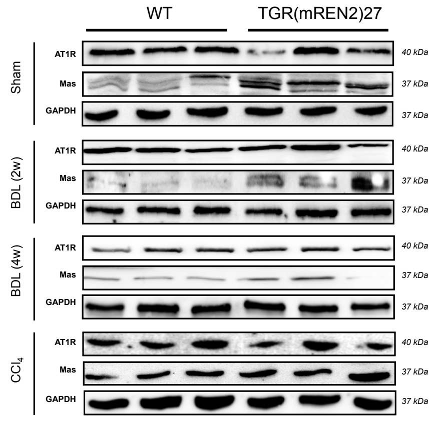


E. Hepatic ACE mRNA expression without and with AVE injection in TGR(mREN2)27 rats

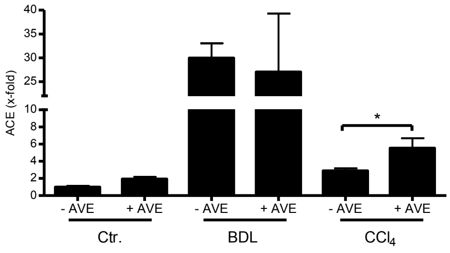


F. Hepatic AT1R mRNA expression without and with AVE injection in TGR(mREN2)27 rats

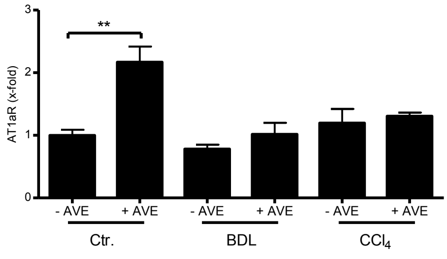


*/**p<0.05/p<0.005

WT TGR(mREN2)27
